# Dilatation de bronches localisée révélant une tumeur carcinoïde

**DOI:** 10.11604/pamj.2016.24.278.9877

**Published:** 2016-07-28

**Authors:** Hind Janah, Hasna Jabri, Régis Gothard Bopaka, Wiam El Khattabi, Hicham Afif

**Affiliations:** 1 Service des Maladies Respiratoires, Hôpital 20 Août 1953, CHU Ibn Rochd, Casablanca, Maroc

**Keywords:** Tumeur carcinoïde, syndrome bronchique récidivant, dilatation des bronches, Carcinoid tumor, recurrent bronchial syndrome, bronchiectasis

## Abstract

Nous rapportons l'observation d'une patiente âgée de 32 ans, qui a présenté un syndrome bronchique purulent récidivant depuis 05 ans. Le bilan radiologique a montré des clartés finement cerclés au niveau basithoracique gauche en rapport avec des dilatations de bronche localisée au niveau du lobe inferieur gauche, de type cylindrique. La bronchoscopie souple a montré une tumeur rougeâtre à surface lisse obstruant l'entrée de la pyramide basale gauche. La patiente a bénéficié d'une lobectomie inferieure gauche plus curage ganglionnaire médiastinal. Les résultats de l'étude anatomopathologique de la pièce opératoire sont en faveur d'une tumeur carcinoïde typique. La suite post-opératoire était sans complication et l'évolution clinique et radiologique est bonne.

## Abstract (en)

We here report the case of a 32-year old female patient with a 5-year history of recurrent purulent bronchial syndrome. Radiological assessment showed a left basithoracic finely ringed bright areas related to cylindrical bronchiectasis located in the left lower lobe. Flexible bronchoscopy showed smooth surface, reddish tumor obstructing the entrance of the left basal pyramid. The patient underwent left lower lobectomy associated with mediastinal lymph node dissection. Histological examination of the surgical specimen was in favor of typical carcinoid tumor. Postoperative outcome was uncomplicated and clinical and radiological evolution was good.

## Introduction

Les bronchectasies sont définies par une augmentation permanente et irréversible du calibre des bronches. Leurs fonctions sont altérées dans des territoires plus ou moins étendus. Les mécanismes physiopathologiques intervenant dans la genèse de la maladie et sa pérennisation font intervenir des facteurs infectieux, mécaniques, environnementaux, toxiques ainsi que des facteurs liés à l'hôte [1, 2]. Cette maladie est fréquente, s'observe chez des patients de plus de 50 ans dans 75% des cas et prédomine chez la femme. Les tumeurs carcinoïdes représentent environ 1 à 2% des cancers broncho-pulmonaires chez l'adulte. Elles touchent le poumon dans près de 10 à 30% des cas [1]. Ce sont des tumeurs à évolution lente qui peuvent être à l'origine de dilatation des bronches (DDB) localisé.

## Patient et observation

**Figure 1:**
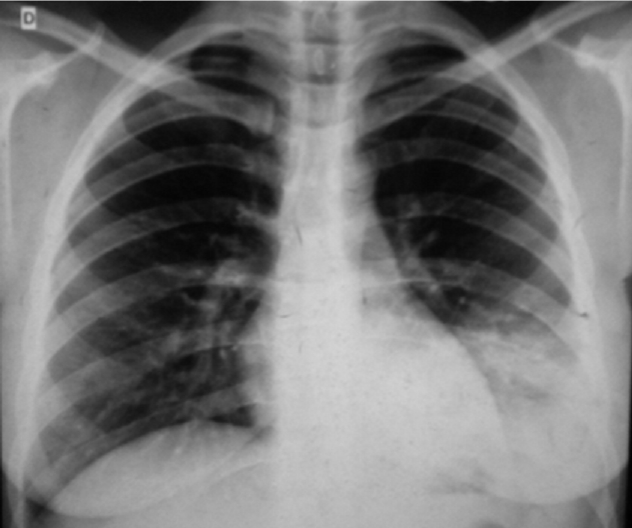
Radiographie thoracique de face: opacité alvéolaire basithoracique gauche

**Figure 2:**
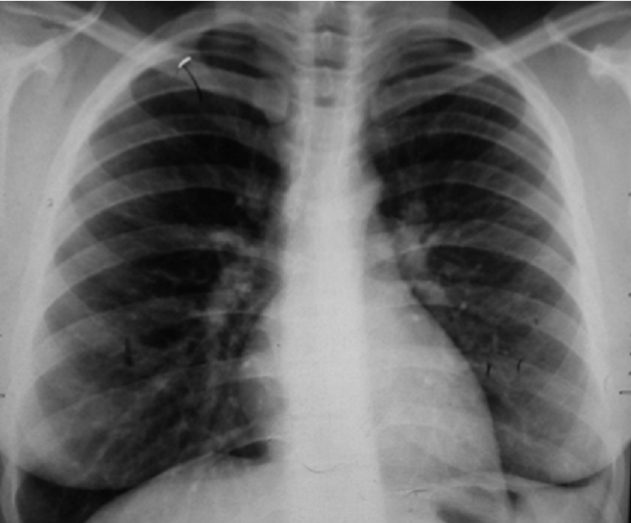
Radiographie thoracique de face: clartés finement cerclés au niveau basithoracique gauche: ascension de la coupole diaphragmatique gauche en rapport avec la lobectomie

**Figure 3:**
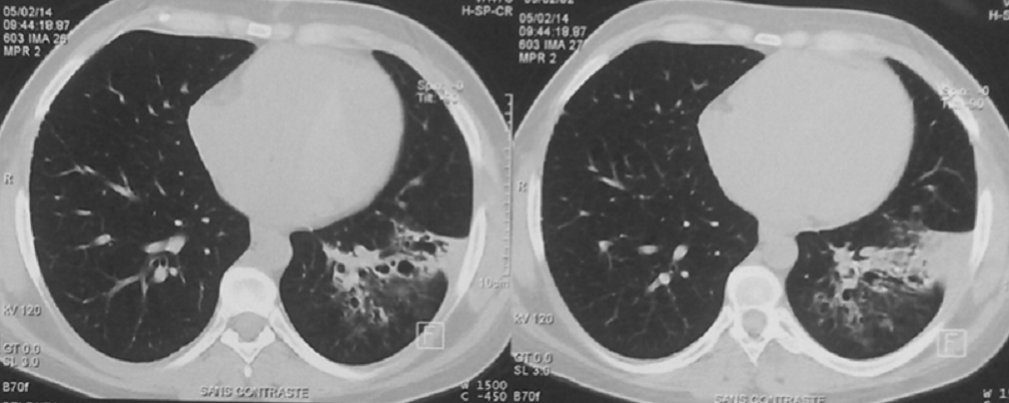
TDM thoracique: dilatation des bronches localisée de type cylindrique au niveau du lobe inferieur gauche

**Figure 4:**
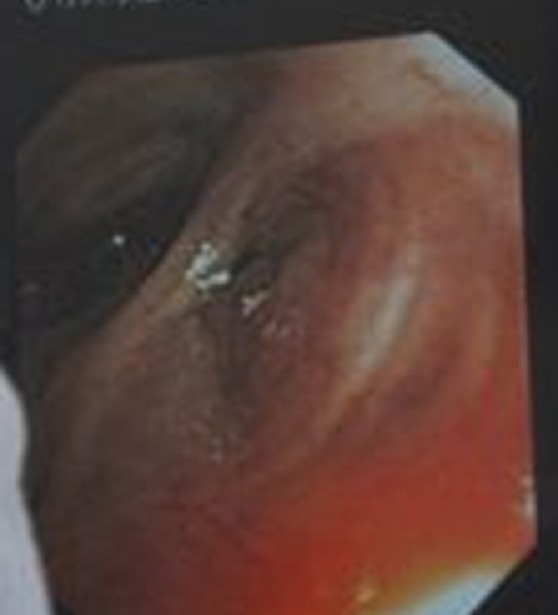
Bronchoscopie souple: bourgeon d’allure tumoral rougeâtre à surface lisse obstruant l’entrée de la pyramide basale

**Figure 5:**
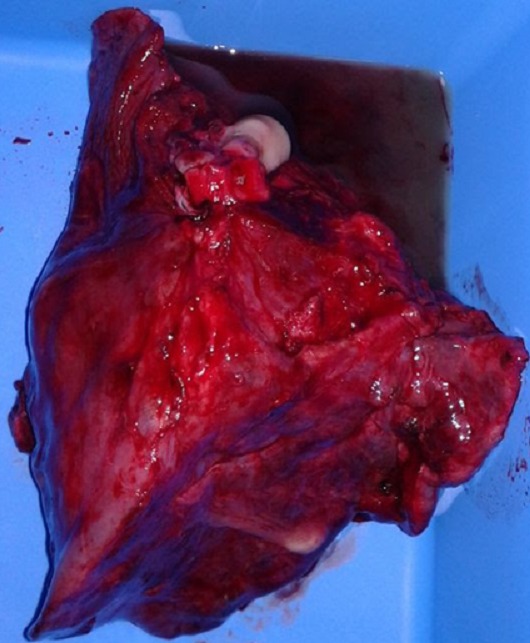
Pièce opératoire d’une lobectomie du lobe inférieure gauche

**Figure 6:**
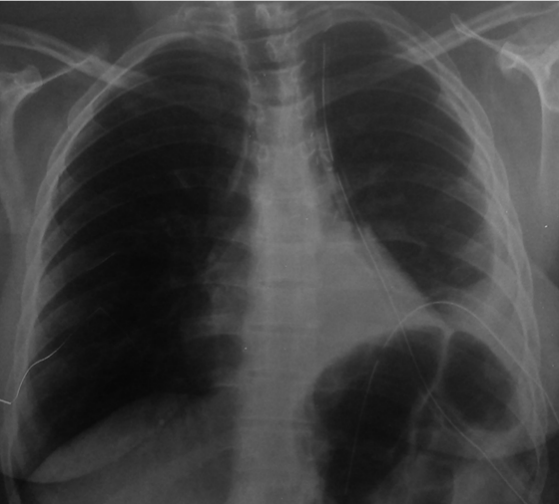
Radiographie thoracique post-opératoire

Mme K.A âgée de 32 ans, mariée et mère de 3 enfants. Elle n'a pas d'antécédents pathologiques particuliers. Elle s'est présentée à la consultation de pneumologie pour syndrome bronchique fait de toux productive ramenant des expectorations purulentes récidivantes malgré des antibiothérapies adaptées à chaque épisode depuis 05 ans, associé à des hémoptysies de moyenne abondance et des douleurs basithoraciques gauches, le tout évoluant dans un contexte de conservation de l'état général. La patiente a bénéficié d'une radiographie du thorax qui a montré une opacité alvéolaire basithoracique gauche (Figure 1). Une antibiothérapie à base d'amoxiciline acide clavulinique a été démarré chez la patiente, dix jours plus tard, nous avons noté la régression de l'opacité alvéolaire laissant place à des clartés finement cerclés au niveau basithoracique gauche évoquant la DDB localisée (Figure 2). Un bilan étiologique ainsi qu'un bilan de retentissement ont été entamé chez la patiente. La tomodensitométrie (TDM) thoracique a confirmé la DDB localisée de type cylindrique au niveau du lobe inférieur gauche (Figure 3). Dans le cadre du bilan étiologique, une bronchoscopie souple a montré un bourgeon d'allure tumoral rougeâtre à surface lisse obstruant l'entrée de la pyramide basale empêchant l'exploration au-delà (Figure 4). La biopsie de la tumeur a été évitée par prudence vue son aspect très hémorragique. Les biopsies de l'éperon lobaire et la carène ont mis en évidence un remaniement fibro-inflammatoire bronchique non spécifique et le liquide d'aspiration bronchique était inflammatoire. Le bilan fonctionnel respiratoire et le reste du bilan préopératoire étaient sans particularité. Dans un but diagnostique et thérapeutique, la patiente a été adressée au service de chirurgie thoracique où une lobectomie inférieure gauche avec curage ganglionnaire médiastinal a été réalisée (Figure 5). Les résultats de l'étude anatomopathologique de la pièce opératoire a montré un foyer d'infarcissement avec nécrose tumorale avec index mitotique inférieur à 1 mitose par champs/10 champs et KI67 moins de 2%, ce qui est en faveur d'une tumeur carcinoïde typique. La suite post-opératoire (Figure 6) était sans complication et l'évolution clinique et radiologique était normale.

## Discussion

Les dilatations de bronches sont des pathologies pouvant résulter de causes multiples, qui peuvent influer sur le traitement et le pronostic. Les DDB localisées nécessitent la recherche d'un facteur mécanique local endobronchique ou extra bronchique. La TDM thoracique permet de distinguer entre dilatations de bronches localisés et diffuses. Cette distinction anatomique est corrélée à des étiologies différentes [1]. Trois étiologies peuvent être en cause dans la survenue de DDB localisées. L'inhalation d'un corps étranger, les adénopathies compressives et en dernier les tumeurs qui sont usuellement bénignes ou à malignité réduite comme le cas de notre observation [2, 3].

Les tumeurs carcinoïdes bronchiques sont peu fréquentes, elles représentent 1 à 2% de toutes les néoplasies pulmonaires des tumeurs développées au dépend des voies aériennes. Elles dérivent des cellules de Kulchitzky de la muqueuse bronchique. On les distingue en deux formes différentes : carcinoïdes typiques qui représentent 80 à 90% des cas et atypique qui représentent 10 à 20% restant. Cette distinction est importante sur le plan pronostique puisque la survie à 10 ans après chirurgie est de 92% pour les carcinoïdes typiques et de 64% pour les carcinoïdes atypiques [4, 5]. Les tumeurs carcinoïdes bronchiques sont des tumeurs à croissance lente. Elles font partie du groupe de tumeur neuroendocrine [6].

Au sein de l'ensemble des tumeurs carcinoïdes, les localisations bronchiques représentent 25% des topographies, c'est le cas de notre patiente. Selon les séries, 20 à 50% des cas sont asymptomatiques et de découverte fortuite. Dans les formes symptomatiques, le délai entre les premiers signes et le diagnostic peut être long. La majorité des patients est symptomatique et peut alors se présenter avec une toux chronique, des douleurs thoraciques, une dyspnée, des hémoptysies, de la fièvre, un wheezing unilatéral ou des infections à répétition. La symptomatologie est d'autant plus parlante que la tumeur est proximale. Dans près de 1 à 5% des cas, un syndrome carcinoïde peut être observé et est secondaire à la libération systémique de substances vasoactives, en particulier la sérotonine (5-HT) [7]. La présentation radiologique, comme la présentation clinique est différente selon que la forme est centrale ou périphérique. Leur point commun est qu'il s'agit de lésions bien limitées, à caractère hypervasculaire, calcifiées dans 30% des cas [8]. L'aspect endobronchique (formes centrales) des tumeurs carcinoïdes est celui de tumeurs: d'aspect lisse, polyploïde, de couleur rouge cerise. L'endoscopie bronchique permet un bilan topographique et d'extension précis. Les biopsies bronchiques exposent à un risque hémorragique non négligeable et la prudence doit être de mise face à des tumeurs endoscopiquement bien rondes et de couleur framboisée. De plus, la petite taille des prélèvements biopsiques bronchiques posent souvent des problèmes diagnostiques [9]. Sur le plan histologique, les tumeurs carcinoïdes typiques sont faites de cellules uniformes, régulières, d'architecture trabéculaire ou en ilot, le noyau, régulier, est central à chromatine fine, avec un petit nucléole inconstant, le stroma est richement vascularisé et on note de manière inconstante des calcifications. L'élément clé pour distinguer entre la forme typique et atypique repose exclusivement sur l'activité mitotique et la présence de nécrose [10].

Le traitement de choix de ces tumeurs carcinoïdes est chirurgical, il dépend de la présentation de la tumeur, une résection partielle (segmentectomie, résection atypique) est proposée dans le cas de tumeur carcinoïde périphérique, alors qu'une chirurgie radicale (lobectomie ou bilobectomie, résection anastomose voire pneumonectomie) avec curage ganglionnaire systématique est l'apanage des formes centrales [1]. Le pronostic à long terme après un traitement chirurgical est bon. La plupart des tumeurs carcinoïdes ont une évolution bénigne. Il s'agit de tumeur de croissance lente. Certaines, plus agressives, peuvent développer des métastases. Selon les séries, les formes dites typiques métastasent dans 2 à 11% des cas et très rarement à distance [11]. La survie des patients atteints de tumeur carcinoïde dépend essentiellement du type histologique et de la présence de métastases [12].

## Conclusion

La mise en évidence d'une étiologie associée à une dilatation des bronches modifie considérablement la prise en charge des patients selon l'étiologie retrouvée. La recherche doit donc être systématique.
